# Linguistic capacity was present in the *Homo sapiens* population 135 thousand years ago

**DOI:** 10.3389/fpsyg.2025.1503900

**Published:** 2025-03-11

**Authors:** Shigeru Miyagawa, Rob DeSalle, Vitor Augusto Nóbrega, Remo Nitschke, Mercedes Okumura, Ian Tattersall

**Affiliations:** ^1^Department of Linguistics and Philosophy, Massachusetts Institute of Technology, Cambridge, MA, United States; ^2^Biosciences Institute, University of São Paulo, São Paulo, Brazil; ^3^Research Center for Super-Smart Society, Seikei University, Tokyo, Japan; ^4^American Museum of Natural History, Institute for Comparative Genomics, New York, NY, United States; ^5^Department of Linguistics, University of São Paulo, São Paulo, Brazil; ^6^Institute for the Interdisciplinary Study of Language Evolution, University of Zurich, Zurich, Switzerland; ^7^Department of Linguistics, University of Arizona, Tucson, AZ, United States; ^8^Laboratory of Human Evolutionary Studies, Department of Genetics and Evolutionary Biology, University of São Paulo, São Paulo, Brazil; ^9^American Museum of Natural History, Division of Anthropology, New York, NY, United States

**Keywords:** human population genomics, early human population divergences, language evolution, modern human behavior, modern human cognition

## Abstract

Recent genome-level studies on the divergence of early *Homo sapiens*, based on single nucleotide polymorphisms, suggest that the initial population division within *H. sapiens* from the original stem occurred approximately 135 thousand years ago. Given that this and all subsequent divisions led to populations with full linguistic capacity, it is reasonable to assume that the potential for language must have been present at the latest by around 135 thousand years ago, before the first division occurred. Had linguistic capacity developed later, we would expect to find some modern human populations without language, or with some fundamentally different mode of communication. Neither is the case. While current evidence does not tell us exactly when language itself appeared, the genomic studies do allow a fairly accurate estimate of the time by which linguistic capacity must have been present in the modern human lineage. Based on the lower boundary of 135 thousand years ago for language, we propose that language may have triggered the widespread appearance of modern human behavior approximately 100 thousand years ago.

## 1 Introduction

More than any other trait, language defines us as human. Yet there is no clear agreement on when this crucial feature emerged in our evolution. Some who have studied the archaeological record suggest that language emerged in our lineage around 100 thousand years ago (kya) (Tattersall, 2012, 2017, 2018; Wadley, 2021), while others have claimed that some form of language preceded the emergence of modern humans (Albessard-Ball and Balzeau, 2018; Botha, 2020). Indeed, it has been argued [e.g., by Progovac (2016) and Dediu and Levinson (2018)] that language is not uniquely the property of the lineage that produced *H. sapiens*. Here we accept the reasoning of that behaviors compatible with language and the consistent exercise of symbolic thinking are detectable only in the archaeological record of *H. sapiens* (Tattersall, 2012; Berwick et al., 2013; Berwick and Chomsky, 2016), and approach the issue of the antiquity of language in our species by showing that, although it is not yet possible to identify the time when a linguistic capacity emerged, genomic evidence allows us to establish with reasonable certainty the latest point at which it must have been present in early *H. sapiens* populations.

Over the past 15 years, numerous studies have addressed the question of exactly when the first division occurred in the original stem population of early *H. sapiens*. While those studies do not tell us exactly when language emerged, they allow us to make a reasonable estimate of the lower boundary of the possible time range for this key occurrence. *H. sapiens* emerged as an anatomically distinctive entity by about 230kya (Vidal et al., 2022). Sometime after that speciation event, the first division occurred, with all descendant populations of that division having full-fledged language. From this universal presence of language, we can deduce that some form of linguistic capacity must have been present before the first population divergence. If the linguistic capacity had emerged in humans after the initial divergence, one would expect to find modern human populations that either do not have language, or that have some communication capacity that differs meaningfully from that of all other human populations. Neither is the case. The 7,000 or so languages in the world today share striking similarities in the ways in which they are constructed phonologically, syntactically, and semantically (Eberhard et al., 2023).

Genomic studies of early *H. sapiens* population broadly agree that the first division from the original stem is represented today by the Khoisan peoples of Southern Africa (Schlebusch et al., 2012). This conclusion was reached early on Vigilant et al. (1989), Knight et al. (2003), Tishkoff et al. (2007), and Veeramah et al. (2012), and it has more recently been bolstered by studies using newer genomic techniques (Fan et al., 2019; Lorente-Galdos et al., 2019; Schlebusch et al., 2017; Schlebusch et al., 2020; Pakendorf and Stoneking, 2021). The term “Khoisan” refers to a bio-genetic affiliation that is linked both to a proposed ancestor-group and to some modern peoples, living in present-day South Africa, who include modern speakers of the Khoe-Khwadi, Tuu, and Ju-ǂHoan languages that have some genetic affiliation to the first divergence of the human population (Güldemann and Sands, 2009; du Plessis, 2014). It follows that, if we can identify when the first division occurred, we can with reasonable certainty consider that date to define the lower boundary of when human language was present in the ancestral modern human population. Based on the results of studies focusing on whole genome single nucleotide polymorphisms (SNPs), we estimate that this first division occurred at approximately 135kya. ^1^

Huybregts (2017) was the first to attempt to pinpoint the timing of the first division in this way. Although he suggested a date of ~125kya, close to our estimate of ~135kya, his estimate was necessarily based on a fairly narrow set of studies showing a remarkably variable range. The studies he examined ranged from the clearly implausible 300kya (Scally and Durbin, 2012), to 180kya (Rito et al., 2013) and as little as 100kya (Schlebusch et al., 2012). Pakendorf and Stoneking (2021) later listed several studies proposing that the first division was older than 160kya (Fan et al., 2019; Lorente-Galdos et al., 2019; Schlebusch et al., 2020), along with four others, from 140 to 110kya, that overlapped with the range suggested by Huybregts (Gronau et al., 2011; Veeramah et al., 2012; Mallick et al., 2016; Song et al., 2017). Several newer studies now allow us to approach the age of the first division with greater precision.

## 2 Divergence time meta-analysis

The literature was searched with combinations of the terms “Khoisan,” “divergence time,” “DNA” and “molecular clock.” We considered only published literature (excluding biorXiv and thesis documents). We also excluded any publication that we deemed a review of previously published work. In this way we obtained 15 publications that contained time estimates for the divergence time of the Khoisan lineage from other human lineages. Publication dates ranged from 2007 to 2023 (Table 1). For each publication we recorded the upper and lower time estimates of divergence, the sample size (both number of individuals and number of populations used, when possible), whether the marker used was maternal (mtDNA), paternal (Y chromosome) or autosomal. In addition, we briefly recorded the informatics methodology that was used to make the estimates. We use a median value of divergence time (Table 2) to summarize the 15 studies for age of the African lineages.

**Table 1:** Summary of estimates of divergence times for Khoisan lineage.

Study	Year	Up	Low	Method	*N*	Marker
Gonder et al.	2007	110	70	r8s	>250	mt
Behar et al.	2008	150	90	PAML/HKY method	624	mt
Gronau et al.	2011	157	108	ABC/mutation rate	6	WG
Veeramah et al.	2012	187	53	ABC/mutation rate	119 i / 8 p	WG
Barbieri et al.	2013	130	115	BEAST/mutation rate	500	mt
Poznik et al.	2013	156	120	Bayes with mutation rate	69	Y
Mallick et al.	2016	173	82	MSMC2*/ mutation rate	300 i/ 142 p	WG
Barbieri et al.	2016	206	178	BEAST/counting mutations	547	Y
Song et al.	2017	141	121	PSMC/related to MSMC	8f	WG
Fan et al.	2019	120	100	MSMC*	92 i/44 p	WG
Lorente-Galdos et al.	2019	180	101	ADMIXTOOL	21 i/15 p	WG
Bergström et al.	2020	162	110	MSMC2*/mutation rate	929/26f	WG
Schlebusch et al.	2020	210	176	MSMC2*/ mutation rate	25 i/ 5 p	WG
Naidoo et al.	2020	133	116	Bayes with mutation rate	19	Y
Ragsdale et al.	2023	135	119	MSMC	add 44 Nama	WG

The first author of each publication is given along with the year of the publication. Divergence times are given in thousands of years. Methods indicate the computational approach to estimation of divergence time. Abbreviations and references in this column are r8s (Sanderson, 2003); PAML/HKY PAML program using HKY model (Yang, 2007); ABC is approximate bayes computation (Csilléry et al., 2010); BEAST (Drummond and Rambaut, 2007); MSMC and a later versions MSMC2 and PSMC are approaches using multiple sequentially Markovian coalescent (Wang et al., 2020); ADMIXTOOL (Petr et al., 2019) is a program used to measure admixture of populations. For sample sizes, some studies reported the number of individuals (i) and the number of populations (p), and whether the genome information was phased. Y is Y chromosomal, mt is mitochondrial and WG is whole genome.

**Table 2:** Median estimates for Khoisan divergence.

Source	Upper	Lower	Median
WG	162	108	135
mt	130	90	110
Y	156	120	138
Non-maternal	159.5	113	136
All	156	110	133

WG indicates median estimate from whole genomes; mt indicates estimates from mtDNA sequences; Y indicates estimates made from Y-chromosomal sequences; non-maternal are estimates from WG plus Y-chromosomal; All are median values for all of the studies shown in Table 1. The median non-maternal values all hover at about 135kya.

### 2.1 Results

To attempt to bring more clarity to the question, we looked at a significantly larger body of work than was available to Huybregts. Estimates of the divergence times of early human populations using molecular markers have been made since the 1980s, when molecular data first began to be available for human evolution research (Soodyall and Jenkins, 1992; Barbujani, 1997; Scozzari et al., 1999; Seielstad et al., 1999; Harris and Hey, 1999; Zhivotovsky et al., 2003). Most of those estimates were made using single genes, or fragments of single genes, or microsatellites. Here we focus on estimates made using whole genome SNPs data, which came onto the scene post-2005. All works are listed in Table 1.

#### 2.1.1 Markers used

These studies vary with respect to the markers used. On the one hand, there are studies using the uniparentally inherited Y chromosome (Y) and mitochondrial DNA (mt) markers; and on the other there are those that use whole genome data. Irrespective of study type, the methods used to determine divergence times are almost exclusively Bayesian, the most precise of them using the recently developed Multiple Sequentially Markovian Coalescent (MSMC) methodology (otherwise known as a “stairway” approach, since it results in a diagram that resembles a stairway). Table 1 summarizes 15 studies (three mt, three Y, and nine whole genome). With the caveats that the estimates of divergence times in those studies were made both with different markers and using different statistical methods for time estimation, we can use them to make an upper and lower boundary for the Khoisan divergence event. Upper estimates of the initial Khoisan divergence from other populations range from 110kya to 210kya, while lower estimates range from 53kya to 178kya. And while these distributions overlap considerably, we can make a fairly replicable estimate of divergence from the data in Table 1, using median values to arrive at ranges and an overall estimate for the divergence time.

#### 2.1.2 Median estimates

The median estimates of divergence for the Khoisan lineage are shown in Table 2. The estimate from whole genome comparisons alone has a median value of 136kya (±23kya). The discrepancy previously noted between male and female lineage divergences (Wilder et al., 2004; Lippold et al., 2014) is also observed here (mt median divergence = 110kya ± 30kya, versus Y chromosomal median divergence = 138kya ±18kya).

While some imprecision in the molecular clock data cannot be eliminated with current techniques, the agreement of those median estimates based on non-maternal markers is clear. It is reasonable from these median estimates to conclude that the original divergence of the Khoisan lineage took place at about 135kya (± 20kya), with the divergence times of other African language lineages lying subsequent to this time.

## 3 Language as a trigger for modern human behavior

These genomic studies of early *H. sapiens* suggest that linguistic potential must have been present in the *H. sapiens* population at the latest by 135kya.

At present, we cannot go back further to pinpoint the date by which language itself emerged. What we can do is to look forward and see how, subsequent to 135kya, language may have had a direct hand in shaping modern human behaviors. To do this we focus on the widespread appearance of suggestive symbolically mediated behaviors, such as the Blombos cave ochres with regular-pattern incisions (Henshilwood et al., 2004; Henshilwood et al., 2009) and the geometric patterns engraved on ostrich eggshells found at two nearby locations, the Diepkloof (Texier et al., 2010; Texier et al., 2013) and Klipdrift Shelters (Henshilwood et al., 2014). While complex behaviors such as burial of the dead and occasional bodily decoration do appear to have occurred sporadically among Neanderthals and other extinct hominins (Frayer et al., 2006; García-Diez et al., 2013; Rodriguez-Vidal et al., 2014; Peresani et al., 2014; Joordens et al., 2015; Radovčić et al., 2015; Hoffmann et al., 2018; Majkić et al., 2018a, 2018b), it appears to have been only within *H. sapiens*, and subsequent to about around 100kya, that such behaviors eventually became systematized across the population (Tattersall, 2012, 2017, 2018; Wadley, 2021). The indicators concerned include such features as the use of pigments (Henshilwood et al., 2011), the employment of pierced marine shells for ornaments and body decorations (Henshilwood et al., 2004; Vanhaeren et al., 2006; d’Errico et al., 2009a), the engraving of non-figurative motifs, complex technologies (Powell et al., 2009; Grove, 2016) and ultimately the earliest representational objects (Henshilwood et al., 2018).

While we find sporadic occurrences of analogous behaviors somewhat earlier in human evolution, and in different contexts (Rodriguez-Vidal et al., 2014; Peresani et al., 2014; Joordens et al., 2015; Radovčić et al., 2015; Hoffmann et al., 2018; Li et al., 2019), it is only after around 100kya that we see such behaviors become routine and normalized in *H. sapiens* (Tattersall, 2012, 2017, 2018; Wadley, 2021). This suggests that linguistic capacity was fully in place before the widespread and normalized appearance of modern human behavior.

What might the role of language have been in the formation and spreading of modern human behavior? Huybregts (2017) argues that, while the linguistic capacity was present in *H. sapiens* prior to 125kya, it only existed as a system strictly internal to our mind, as suggested by Chomsky (2010, 2016), and did not become externalized as a communication system until sometime later, which puts it in the timeline of those other modern human behaviors. The ~77kya Blombos engraved ochres have similarly been considered as proxies for language, and not necessarily to have been preceded by it (Henshilwood and Marean, 2003, 2006; d’Errico et al., 2009b; Henshilwood and Dubreuil, 2009; Henshilwood and d'Errico, 2011). Some have suggested that language is a prerequisite for symbolically-mediated behavior (Henshilwood et al., 2004; Henshilwood and Dubreuil, 2009).

We wish to note the specific role that language may have played in organizing, and hence systematizing, modern behavior. Our proposal is similar to earlier suggestions by Henshilwood and others, but is based on a concrete and verifiable date of approximately 135 kya as the lower boundary for the presence of language. As the most complex communication tool yet devised in nature, it had a direct and enormous impact on all facets of human life. Language, with its complex system of mental representations and rules for combining them, is able to create new ways to connect existing symbols and predict new ways of behavior. This is, perhaps, what we see in the time gap between the lower boundary of 135kya for language, and the beginnings of the emergence of rich and normative symbolic behavior starting around 100kya. A way to interpret this gap is that language was central in organizing and systematizing modern human behavior.

As an alternative to the view that language was the trigger for modern human behavior (Tattersall, 2012, 2018), a number of archaeologists favor the view of an incremental, cumulative assembly of such modern human behavior as symbolic material culture, technological diversification, the use of diverse raw materials, and use of extensive social networks (Scerri and Will, 2023). One way to combine this view with ours is to consider language as the trigger that both accelerated and consolidated all these gradual processes that were taking place over the Middle Stone Age. One clear point of agreement with these scholars is that all believe that modern human behaviors emerged within Middle to Late Pleistocene in Africa.

## 4 The picture that emerges

Based on the recent genetic studies of early *H. sapiens*, we have pinpointed approximately 135kya as the moment at which some linguistic capacity must have been present in the human population. Looking forward from this event, modern human behaviors such as body decoration and the production of ochre pieces with symbolic engravings appeared as normative and persistent behaviors around 100kya. We believe that the time lag implied between the lower boundary of when language was present (135kya) and the emergence of normative modern human behaviors across the population suggests that language itself was the trigger that transformed nonlinguistic early *H. sapiens* (who nonetheless already possessed “language-ready” brains acquired at the origin of the anatomically distinctive species) into the symbolically-mediated beings familiar today. This development of the most sophisticated communication device in evolution allowed our ancestors to accelerate and consolidate symbolically-mediated behaviors until they became the norm for the entire species.

## Data Availability

Publicly available datasets were analyzed in this study. This data can be found at: the data utilized in this manuscript is available in the set of articles explored. In the manuscript, you can find all relevant references to the publications where the data is presented.

## Footnotes

^1^It is important to note that in the current debate on human language evolution, at least two major perspectives are considered across different disciplines engaged with this topic. The first is the gradualist view, which sees the modern complexity of human language as the result of an incremental evolutionary process (Pinker and Bloom, 1990; Jackendoff, 1999, 2002; Tallerman, 2014, 2017; Progovac, 2015, Martins and Boeckx, 2019, a.o.). The second is the saltational view, which posits that most components of present-day linguistic capacity were exapted and assembled in a sudden, catastrophic event, giving rise to a cognitive faculty whose complexity did not emerge from gradual tinkering over eons (Bickerton, 1990, 1998; Berwick, 1998; Chomsky, 2002, 2005, 2016; Di Sciullo, 2013; Nóbrega and Miyagawa, 2015; Watanabe et al., 2017; Berwick and Chomsky, 2016, 2019, 2011, a.o.). For a comprehensive overview of these two perspectives, see Progovac (2019). The empirical implications of this article, however, do not allow for a definitive determination of which approach is on the right track. Rather, it provides insight into the earliest possible boundary by which language was available, without necessarily committing to the specific evolutionary changes that led to its emergence. In any case, any proposal suggesting that linguistic capacity emerged later than 135kya (e.g., Klein and Edgar, 2002; Chomsky, 2005) appears, in our view, highly implausible.
